# miR-138 exerts anti-glioma efficacy by targeting immune checkpoints

**DOI:** 10.1186/2051-1426-1-S1-P177

**Published:** 2013-11-07

**Authors:** Jun Wei, Edjah Nduom, Ling-Yuan Kong, Fei Wang, Shuo Xu, Konrad Gabrusiewicz, Ali Alum, Greg Fuller, George Calin, Amy B Heimberger

**Affiliations:** 1 University of Texas MD Andrson Cancer Center, Houston, TX, USA; 2 Qilu Hospital of Shandong University, Jinan, China

## 

The immune checkpoints, CTLA-4 and PD-1, are negative regulators of T cell activation and are inducers of FoxP3+ Tregs. Monoclonal antibody therapy to each has demonstrated tumor regression in clinical trials and potent therapeutic synergy has been observed when used in combination. MicroRNAs (miRs) have been shown to modulate gene transcripts involved in tumorigenesis and can target tumor-mediated immune suppression. On the basis of differential miRNA gene expression libraries from glioblastoma patients, miR-138 was identified as a top down regulated candidate. On glioma tumor microarrays, miR-138 expression is heterogeneous among the various grades and pathologies. Target binding algorithms predicted that miR-138 could bind CTLA-4 and PD-1. Transfection of human CD4+ T cells with miR-138 suppressed CTLA-4-, PD-1-, and FoxP3- expression in vitro. Treatment of established subcutaneous GL261 murine glioma cells in immune competent C57BL/6 mice with miR-138 or scramble control administered intravenously demonstrated that gliomas started to shrink as soon as miR-138 was administered. Moreover, the gliomas continued to regress even after miR-138 treatment was discontinued. In contrast, tumors kept growing aggressively in scramble miRNA-treated and untreated tumor-bearing mice groups. Furthermore, in C57BL/6 mice with established intracerebral GL261 treated with i.v. administered miR-138, median survival was 33.5 days relative to mice treated with scramble control with a median survival of 23.5 days (P=0.011). Intravenous treatment of mice with established intracerebral gliomas with miR-138 relative to the scramble control reduced the relative incidence of Tregs by 51% (P=0.03) within the glioma microenvironment. Formulation equivalency studies in vivo indicate that DOTAP causes rapid translocation of miRNAs into the peripheral blood compartment indicating that miR-138 may have rapid translational potential as a novel immunotherapeutic agent for neoplasms.

